# Delineating a Retesting Zone Using Receiver Operating Characteristic Analysis on Serial QuantiFERON Tuberculosis Test Results in US Healthcare Workers

**DOI:** 10.1155/2012/291294

**Published:** 2012-12-30

**Authors:** Wendy Thanassi, Art Noda, Beatriz Hernandez, Jeffery Newell, Paul Terpeluk, David Marder, Jerome A. Yesavage

**Affiliations:** ^1^Department of Medicine, Veterans Affairs Palo Alto Health Care System, 3801 Miranda Avenue MC-, Palo Alto, CA 94304-1207, USA; ^2^Occupational Health Strategic Health Care Group, Office of Public Health, Veterans Health Administration, Washington, DC 20006, USA; ^3^Division of Emergency Medicine, Stanford University School of Medicine, Stanford, CA 94304, USA; ^4^War Related Illness and Injury Study Center (WRIISC) and Mental Illness Research Education and Clinical Center (MIRECC), Department of Veterans Affairs, Palo Alto, CA 94304, USA; ^5^Department of Psychiatry and Behavioral Sciences, Stanford University School of Medicine, Stanford, CA 94304, USA; ^6^Department of Occupational Health, The Cleveland Clinic, Cleveland, OH 44195, USA; ^7^University Health Services, University of Illinois Chicago, Chicago, IL 60612, USA

## Abstract

*Objective*. To find a statistically significant separation point for the QuantiFERON Gold In-Tube (QFT) interferon gamma release assay that could define an optimal “retesting zone” for use in serially tested low-risk populations who have test “reversions” from initially positive to subsequently negative results. *Method*. Using receiver operating characteristic analysis (ROC) to analyze retrospective data collected from 3 major hospitals, we searched for predictors of reversion until statistically significant separation points were revealed. A confirmatory regression analysis was performed on an additional sample. *Results*. In 575 initially positive US healthcare workers (HCWs), 300 (52.2%) had reversions, while 275 (47.8%) had two sequential positive tests. The most statistically significant (Kappa = 0.48, chi-square = 131.0, *P* < 0.001) separation point identified by the ROC for predicting reversion was the tuberculosis antigen minus-nil (TBag-nil) value at 1.11 International Units per milliliter (IU/mL). The second separation point was found at TBag-nil at 0.72 IU/mL (Kappa = 0.16, chi-square = 8.2, *P* < 0.01). The model was validated by the regression analysis of 287 HCWs. *Conclusion*. Reversion likelihood increases as the TBag-nil approaches the manufacturer's cut-point of 0.35 IU/mL. The most statistically significant separation point between those who test repeatedly positive and those who revert is 1.11 IU/mL. Clinicians should retest low-risk individuals with initial QFT results < 1.11 IU/mL.

## 1. Introduction

We report the findings of a multisite study of United States healthcare workers (HCWs) that began as a quality control initiative in the Veterans Administration Palo Alto Health Care System (VAPAHCS) when QuantiFERON Gold In-Tube (QFT) serial screening tests were observed to be initially positive and were subsequently negative in those low-risk individuals. This seemingly spontaneous “reversion” has been reported around the world in the literature, and the variability that occurs mostly around the baseline is recognized [1–6]. 

This study design was driven by the clinical experience: when an HCW presents with a positive QFT result, what can the clinician do to discern whether the next test is likely to remain positive or become negative? 

The foundation of the problem lies in the dichotomous nature of the results reported. Currently, a QuantiFERON tuberculosis antigen minus-nil (TBag-nil) ≥ 0.35 International Units per milliliter (IU/mL) is reported as “positive.” At that point the provider has a decision to make, one that is generally to investigate further with a chest radiograph, seek specialty consultation, and/or recommend medical treatment. Whereas positive tuberculin skin tests (TSTs) were often felt to be erroneous due to prior BCG vaccination, and compliance and treatment rates were low; studies are showing that positive interferon-gamma release assay (IGRA) results are more likely to lead to both the recommendation and the acceptance of chemotherapy [7–10]. Chemotherapy puts the patient at risk for side effects including hepatotoxicity [11], as well as social stigma or workplace discrimination [12]. The presumptive diagnosis of tuberculosis infection in HCWs, particularly when interpreted as an occupational conversion, can trigger Occupational Safety and Health Administration, National Institute for Occupational Safety, and Health or hospital infection control contact investigations that are both time consuming and costly. Thus the presence of spontaneous “reversions” implies that clinicians and patients are experiencing unnecessary concern, action, or expense and potentially placing patients in harm's way for transiently positive results which are forced by the binary nature of the current reporting structure. There is a need for increased accuracy and efficiency in the screening process to reduce the burdens to the patient and the system, and utilization of this predictive tool may lend some assistance.

In response to the persistent concerns regarding reversions near the cut-point of 0.35 IU/mL, a 2010 Morbidity and Mortality Weekly Report published by the Centers for Disease Control and Prevention (CDC) recommended that quantitative QFT results should be reported. The CDC did not, however, provide guidance for either the interpretation or the use of these values [13]. We investigated reversions in US HCWs in order to develop a validated model, using receiver operating characteristic analysis, to define the range of results that best predicts a transiently positive result. With the ability to predict the likelihood of reversion, clinicians and patients could choose to retest rather than to pursue costly and time-consuming consultations and therapies.

## 2. Materials and Methods

### 2.1. Participants and Variables

Data were obtained from a retrospective review of available clinical laboratory records from three different sites: (1) Veterans Administration Palo Alto Health Care System (VAPAHCS), California, (2) University of Illinois Chicago (UIC) Il, and the (3) Cleveland Clinic (CC), Ohio, where each HCW undergoes preemployment and annual QFT testing irrespective of previous results. All subjects are US HCWs who were serially tested by QFT Gold-in-Tube in their hospital's laboratory. All HCWs at least 18 years of age with available records were included. The study's date range was January 2009 through June 2011 at VAPAHCS, from August 2008 through June 2011 at UIC, and from October 2009 through December 2011 at the Cleveland Clinic. 

HCWs who tested consistently negative and those with only a single test result were excluded. Results reported without the QFT TBag-nil numerical value, as well as HCWs with negative-to-positive discordance/conversion at the conclusion of their testing series were removed from the dataset (22/195 from VAPAHCS, 124/742 from UIC, and 61/127 from CC). To be included in the analysis, at least two QFT tests were required, one of which was a positive result that was followed by either a positive or a negative result. This reproduces the clinician's actionable decision point; that is, when a patient presents with a positive result, the action to test further, to refer, or to treat is initiated. Patients were only included once (see Table 1).

### 2.2. Participant Sites

The VAPAHCS is a suburban teaching hospital located in Palo Alto, California. The county in which it resides, Santa Clara, has the 3rd highest tuberculosis (TB) rate in California [14] at 11.4% from 2006–2011 [15], and California is ranked 3rd in USA for TB cases behind Alaska and Hawaii [16]. All VAPAHCS HCWs are United States citizens. The HCW population is approximately 3,500. The lab performed over 16,000 QFT-GIT tests (including patient testing) during this period. Of the 4,019 HCWs who were tested between January 1, 2009 and June 30, 2011, 2,706 (67%) tested negative one time and 293 (7%) tested positive one time, without repeat testing. (Note that VAPA also tests researchers, students, volunteers, and Peace Corps personnel, most of whom are on campus for only one testing cycle). Of the 4,019 unique HCWs, 781 (19%) tested negative more than once and never tested positive. Thus the overall negative rate at VAPA is (67% + 19%) = 86%, while 14% of personnel tested positive at least once in their series. The indeterminate rate in this lab is 0.4%.

The University of Illinois, Chicago (UIC) is a public, urban academic teaching hospital. The HCW population is approximately 5,000. Their laboratory performed over 50,000 QFT-GIT tests by June 2011; 20,543 of these were on HCWs. Annual HCW TB screening is mandated and compliance is 99%, with most HCWs tested annually, but some who are on surveillance are tested every six months. UIC reports a HCW QFT negative rate of 89.5%, with 1.1% indeterminate and 9.4% positive at some point in their series. Illinois ranks 21st for tuberculosis cases in the nation [16], and Chicago itself had a TB incidence rate recorded at 7.4% during 2006–2010 [17].

The Cleveland Clinic Foundation (Cleveland, OH, USA) is an urban teaching hospital. The laboratory had performed over 10,000 QFT-GIT tests by June 2011. This includes patient and HCW testing. Cleveland Clinic hires approximately 2,500 HCWs annually. The HCW population is 98.5% negative with 0.5% indeterminate and 1% positive at some point in their series. Ohio ranks 35th in the nation for tuberculosis cases [16] with Cleveland itself having a 6.4% case rate from 2006–2010 [18]. 

### 2.3. QuantiFERON Gold In-Tube Blood Assay (Qiagen, Inc)

The interferon gamma released was measured by enzyme-linked immunosorbent assay (ELISA) according to the manufacturer's protocol though with an 8-point standard curve for each microplate. The results were read at 450 nm by the Diamedix DS2 Automated ELISA System (Diamedix Corporation, Miami, FL) at VAPAHCS, by the Diamedix DSX Automated ELISA System (Diamedix Corporation, Miami, FL) at UIC, and by a BioTek ELx800 Absorbance Microplate Reader (BioTek Instruments, Winooski, VT) at CC. All tests in this series met the nil, mitogen, and the equation criteria for test validity delineated in the manufacturer's package insert [19].

### 2.4. Measures

In the absence of a gold standard against which to evaluate latent tuberculosis infection, the expected probability of two consecutive positive tests was employed as a proxy for corroboration of the test result in question, which is the implied presence of latent tuberculosis disease. In seeking what would best predict whether an individual was likely to be a “reverter”, the initial TBag-nil value in the series of two sequential tests was evaluated as a possible predictor variable. Note that the QFT result was considered positive if the TBag-nil was ≥0.35 IU/mL, so all TBag-nil values were at least 0.35 IU/mL in this analysis. 

### 2.5. Data Analytic Approach

We used a two-step data analytic approach. First, we employed a receiver operating characteristic analysis (ROC) [20, 21] on two-thirds (the Exploratory Group) of the 862 HCW sample to identify characteristics that might significantly differentiate reversions from those with two consecutively positive results. An ROC analysis is an exploratory process that searches every value of every predictor variable entered to identify the variable and value that results in the highest sensitivity and specificity (using the weighted kappa statistic) for identifying the targeted criterion. The targeted criterion in this case is reversion. Second, because ROC is an exploratory technique, we conducted a confirmatory logistic regression analysis and chi-square tests using the remaining one-third of the HCWs (the Confirmatory Group) to examine whether the predictor that had been identified in the first step did in fact predict reversion in an independent sample. 

Regarding the details of the ROC analysis, once the optimal variable and associated separation point are identified, the association with the success criterion is tested against a stopping rule. Stopping rules include a subgroup sample size too small for further analysis (*n* < 20) and/or when no further variables are selected because the *P* value associated with the Chi-square statistic is ≥0.01. If the association does not meet the criteria for the stopping rule, the sample is divided into two groups based on the optimal variable and identified separation point. The ROC analysis is then restarted, separately, for each of these two subgroups. The result is a decision tree identifying the HCW characteristics and associated separation points that best predict reversions, with *P* values, chi-square, and Kappa values calculated and reported. The ROC software developed by Drs. Yesavage and Kraemer is publicly available [21], and the logistic regression and Chi-square tests were performed using SAS software (Version 9.3, Cary, NC, USA). 

## 3. Results and Discussion

### 3.1. Results

HCWs from each site had undergone between 2 and 9 tests in series. The most recent positive test that was followed by either a positive (no reversion) or negative (reversion) result defined the two test results in the series that were analyzed (see Table 1). The mean number of days between tests was 434 for VAPAHCS, 261 for UIC, and 235 for CC.

**Figure 1:**
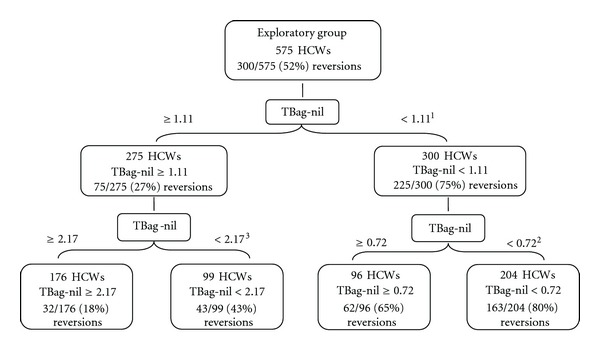
Receiver operating characteristic (ROC) decision tree identifying statistically significant TBag-nil (in IU/mL) separation points which predict those HCWs with a positive TB test result at time one who retest negative at time two. Logistic regression analysis on a separate Confirmatory sample of 287 HCWs validated all 3 separation points at 0.72, 1.11, and 2.17 IU/mL and remained statistically significant for all subgroups by chi-square (*P* < 0.001). 1 Kappa = 0.48, chi-square = 131.0, *P* < 0.001, 2 Kappa = 0.16, chi-square = 8.2, *P* < 0.01, 3 Kappa = 0.27, chi-square = 20.4, *P* < 0.001.

The 862 HCWs who met inclusion criteria were randomly assigned to one of two groups: the Exploratory Group (*n* = 575) or the Confirmatory Group (*n* = 287). The Exploratory Group of tested HCWs had a 52.2% (300/575) reversion rate. The results of the ROC analysis performed on the Exploratory Group are presented as a decision tree shown in Figure 1. TBag-nil in IU/mL was most statistically significant for predicting reversion at the separation point 1.11 IU/mL (Kappa = 0.48, chi-Square = 131.0, *P* < 0.001). Two groups of HCWs were identified:

 group 1: 75% reversions: 225/300 HCWs with TBag-nil <1.11 IU/mL;
 group 2: 27% reversions: 75/275 HCWs with Tbag-nil ≥1.11 IU/mL.

The ROC analysis further identified two subgroups of HCWs derived from group 1 above with a TBag-nil at 0.72 IU/mL (Kappa = 0.16, chi-square = 8.2, *P* < 0.01):

 group a: 80% reversions: 163/204 HCWs with TBag-nil <0.72 IU/mL;
 group b: 65% reversions: 62/96 HCWs with TBag-nil ≥0.72 and <1.11 IU/mL.

Two subgroups of HCWs were also identified from group 2 above with TBag-nil at 2.17 IU/mL (Kappa = 0.27, chi-Square = 20.4, *P* < 0.001):

 group c: 43% reversions: 43/99 HCWs with TBag-nil ≥ 1.11 and <2.17 IU/mL;
 group d: 18% reversions: 32/176 HCWs with TBag-nil ≥ 2.17 IU/mL.

**Figure 2:**
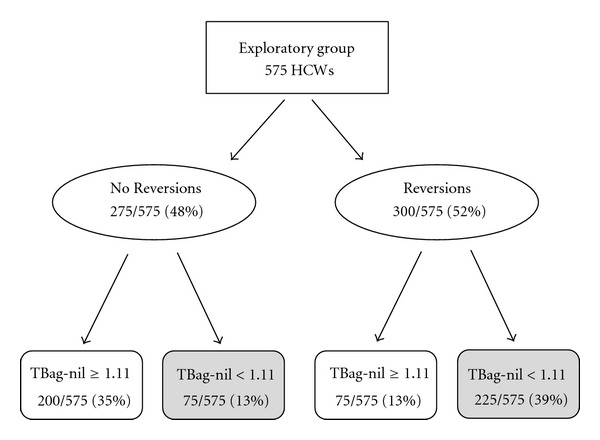
Exploratory group with 575 HCWs classified as No Reversions (those with two positive tests) or Reversions (with a positive TB test result at time one and who retest negative at time two). The two groups are further classified by TBag-nil values using the ROC selected separation point of 1.11 IU/mL (Kappa = 0.48, chi-square = 131.0, *P* < 0.001). Highlighted boxes emphasize the difference in number of No Reversions versus Reversions when TBag-nil < 1.11 IU/mL, the identified retesting zone.

Figure 2 contains a decision tree classifying the 575 HCWs in the Exploratory Group as Reversions or No Reversions and by TBag-nil values using the ROC selected separation point of 1.11 IU/mL. Note that 225 of the 300 “reverters” are identified at this separation point.

A logistic regression analysis was conducted in the Confirmatory Group (*n* = 287) using the same dependent measure (reversion) and predictor variable (TBag-nil) identified in the primary ROC analysis. The relationship remained statistically significant (*P* < 0.001). All three separation points at 0.72, 1.11, and 2.17 IU/mL (4 subgroups) identified in the ROC analysis also remained statistically significant for all subgroups by chi-square (*P* < 0.001). 

## 4. Discussion

Multiple papers have reported within-subject variability in serial QFT results [1, 3, 6, 22, 23], and much work has been done to unmask a retesting zone by suggesting alternative separation points of 0.5, 0.7 [6], or 1.0 IU/mL [23]. In Europe, employing a borderline zone between 0.2–0.7 IU/mL decreased conversions and reversions from 1.9 to 0.6% and from 6.1 to 2.6%, respectively, with no active tuberculosis cases occurring in the “positive” population in a 2-year follow-up period [24].

Further, it is both observed and understood that QFT reversions are much more common than conversions. Among the many studies published and reviewed on this topic [25], Schablon et al. [22] reports a conversion to reversion ratio of 6.1 versus 32.6% in 287 German healthcare workers, which is the same range as studies conducted in the United States (6.3 versus 33%) [26]. 

The predominance of reversions is likely explained in part by the statistical phenomenon of regression to the mean [24]. Regression to the mean (RTM) is the tendency of observations to move closer to the mean when repeated. When measurements are repeated in individuals, and measures are selected based on exceeding an absolute threshold in an inherently continuous range of values, influence by RTM should be considered. Examples of RTM are common in clinical medicine. In this case, since the observed mean result in these US HCWs is <0.35 IU/mL, retesting a population subset that is initially above that mean will likely yield values that are closer to the population mean (in this case, in the negative range). The population “conversion” rate will be a mix of both true incident disease (proportional to the epidemiology of TB in the US HCWs) and false positives that will likely have reversions. The challenge is to identify a retesting zone with an upper value that minimizes noise while still identifying clinically significant cases for followup in a cost-effective manner.

As for the reliability of that negative result, the QFT Gold In-Tube has a specificity of 99% [19], reflecting the measurement of persons correctly identified as not having the condition (in this case tuberculosis). Further, the prevalence of disease in this population is low, making the pretest probability of positive results low. Additionally, Diel et al. conducted a study of 954 persons exposed to active tuberculosis and report a negative predictive value of 99.7% after 5 years [27]. With all of this in mind, the authors conclude that while the decision on how to act upon a test result lays with the clinician and never purely with numerical data, a negative QFT result is significantly more reliable than a low positive result in its ability to predict disease or the lack thereof.

To help clarify a practice algorithm, there is a call in the literature for a statistically based, data-driven retesting zone. Zwerling et al. in a 2011 review article in *Thorax* concluded that “the use of IGRAs for serial testing is complicated by lack of data on optimum cut-offs for serial testing …” [28], and a 2012 editorial in *Chest* stated that “it is quite arbitrary to limit true conversion to those with a QFT-GIT of >1.0 IU/mL, since that value, though a nice round figure, has not been validated” [29]. Here we offer that a statistically driven optimal separation point between consistently positive serially tested US healthcare workers and healthcare workers who are likely to revert is 1.11 IU/mL. 

We focus on 1.11 IU/mL as the border of a retesting zone because it was determined by the Kappa statistic in the ROC software as the optimally sensitive and specific separation point between the “reversions” from those who did not “revert” in this multisite cohort. At a separation point of 1.11 IU/mL, sensitivity is 0.75 and specificity is 0.73, whereas at a separation point of 0.72 IU/mL sensitivity is 0.54 and specificity is 0.85. The respective Kappas are 0.48 versus 0.39. As is the case by lowering the retesting separation point to 0.72 IU/mL, further lowering it to 0.50 IU/mL would increase the specificity of the measure to 0.93 and capture 84/103 (82%) of reversions in this range, but this would include only 103/575 (18%) of the total population and 84/300 (28%) of the “reverters.” The sensitivity of this separation point would be only 0.28 and its Kappa 0.21. Thus with the ROC there are trade-offs in sensitivity versus specificity, depending upon the separation points selected. The 1.11 IU/mL measurement was chosen by the ROC analysis for this population because that value maximizes the percentage of “reverters” while optimizing sensitivity and specificity.

## 5. Conclusion

We present a validated model on a sample of 862 US healthcare workers from three major US hospitals that could be used to define a QuantiFERON Gold In-Tube retesting zone between 0.35 and 1.11 IU/mL. The upper value was selected by a receiver operating characteristic analysis to maximize separation between HCWs who have two consecutive positive tests and those who have reversions (*P* < 0.001). Our sample of HCWs had a 75% risk for reversion if their initial positive test fell within this range. While 0.35–1.11 IU/mL is therefore the optimal retesting zone identified here, 0.35–0.72 IU/mL (80% reversion; *P* < 0.01) is another possible separation point also selected by the ROC and could be reasonably employed by providers based on the clinical situation much like the 5, 10, and 15 mm tuberculin skin test cut-off points that are used in different settings. Acceptance of TBag-nil values reported above as the delineators of a QFT retesting zone could lessen patient anxiety, decrease unnecessary radiographs, prevent unnecessary exposure investigations, and possibly spare patients from inappropriate medical treatment due to transiently “positive” QFT test results. 

## 6. Limitations and Future Directions

Limitations of the study include that while the current analyses incorporated over 850 positive HCW records, these data are from only three facilities. Furthermore, results in this study are weighted towards UIC, since their data comprise the majority of the sample group. While there could be selection bias among those HCWs who present for serial testing, it is not clear how that could influence these results. Finally, it should also be noted that prospective long-term followup would be required to provide thorough validation of the results. 

Future analyses using the same statistical methods could include additional data from other institutions in USA, Europe, or from countries with higher risk for HCWs. There is a possibility that there could be local variation based on biologic or regional laboratory differences that would be exposed when more data are analyzed. 

## Figures and Tables

**Table 1:** Test results for analyzed HCWs from VA Palo Alto Health Care System (VAPAHCS), University of Illinois Chicago (UIC) and the Cleveland Clinic (CC).

Test results	VAPAHCS	UIC	CC	Total (*n*)
Repeat positive result	113	338	25	476
Reversion	73	275	38	386

Total (*n*)	186	613	63	862

Note: HCWs were excluded if their only positive test result was their last test taken or if data for only one test result was available.
